# Molecular Derailment via Pressurization in Methylammonium Lead Iodide

**DOI:** 10.1021/acs.jpclett.5c01832

**Published:** 2025-10-13

**Authors:** Pelayo Marin-Villa, Pablo Gila-Herranz, Monica Jimenez-Ruiz, Alexandre Ivanov, Jeff Armstrong, Kacper Drużbicki, Felix Fernandez-Alonso

**Affiliations:** † Centro de Física de Materiales (CFM-MPC), CSIC-UPV/EHU, Paseo de Manuel Lardizabal 5, Donostia 20018, Gipuzkoa, Spain; ‡ 56053 Institut Laue Langevin, F-38042 Grenoble, France; ¶ 120797 ISIS Neutron and Muon Facility, Rutherford Appleton Laboratory, Didcot OX11 0QX, United Kingdom; § Polish Academy of Sciences, 86897 Centre of Molecular and Macromolecular Studies, Sienkiewicza 112, 90-363 Lodz, Poland; ∥ Donostia International Physics Center (DIPC), Paseo de Manuel Lardizabal 4, 20018 Donostia-San Sebastian, Spain; ⊥ IKERBASQUE, Basque Foundation for Science, Plaza Euskadi 5, 48009 Bilbao, Spain

## Abstract

Hybrid organic–inorganic perovskites combine outstanding optoelectronic properties with low-cost fabrication, yet their structural fragility under environmental factors limits device stability. In this context, the use of pressure offers the enticing possibility of unveiling the microscopic mechanisms behind structural changes and the eventual collapse or decomposition of the material. In this work, we have employed high-resolution inelastic neutron scattering in the gigapascal regime alongside first-principles calculations to probe the pressure–temperature phase behavior of methylammonium lead iodide (MAPbI_3_). Below 1 GPa and 150 K, pressurization leads to a stiffening of spectral features sensitive to NH···I hydrogen-bonding motifs, concomitant with a contraction of the inorganic framework. Between 1 and 1.25 GPa at these low temperatures, the INS data undergo a pronounced broadening, corresponding to the formation of an orientational glass of organic cations whose immediate environment is reminiscent of the high-pressure cubic phase. This hitherto unexplored *derailed* state of MAPbI_3_ is characterized by a broad distribution of NH···I bond lengths, in stark contrast with the well-defined hydrogen-bond network of the low-temperature phase observed at lower pressures. Our experimental and computational results bring to the fore the rather subtle role played by the NH···I bonding topology across organic and inorganic sublattices in dictating the regions of physical stability and metastability of this important material.

## Graphical abstract

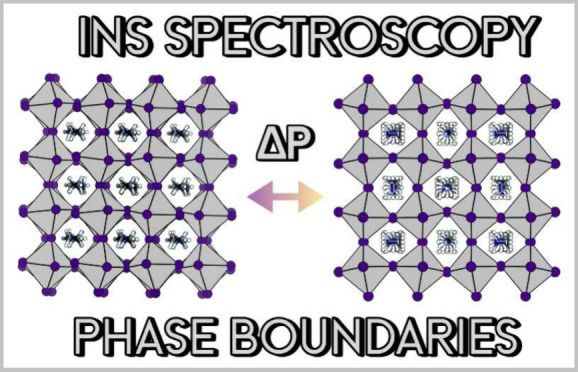

Current interest in Hybrid Organic–Inorganic Perovskites (HOIPs) as promising candidates for photovoltaics, photonics and other emerging applications stems from their superb optoelectronic properties and low fabrication costs.^1,2^ Notwithstanding these merits, a number of both intrinsic and extrinsic limitations still hinder the full deployment of HOIPs in technological devices.^3^ The fragility of the perovskite framework in the presence of environmental factors such as moisture or UV radiation leads to various degradation mechanisms of the crystalline structure, ranging from the appearance of structural imperfections to complete phase decomposition.^3^ Continued efforts to suppress the sensitivity of active layers to external agents have resulted in several successful strategies for surface passivation and encapsulation, leading to improved durability and performance of HOIP-based devices.^4^ Despite these achievements, ion migration and degradation of the perovskite framework continue to pose major challenges for their commercial deployment.^4,5^ Overcoming these obstacles calls for a comprehensive exploration of HOIPs at the microscopic level, in order to understand and ultimately control the atomic and molecular mechanisms underpinning stabilization and destabilization pathways. In this context, our most recent study using a combination of diffraction (neutron and synchrotron) and first-principles calculations across the phase diagram of MAPbI_3_ has yielded new insights on the mechanisms at play ultimately leading to structural collapse when accessing the GPa range.^6^ By circumventing chemical modifications, physical pressure serves as a valuable tool to probe structural stability and lattice distortions, while simultaneously providing a means of tuning structural and optical properties.^7,8^ These efforts further contribute to the development of more effective strategies to overcome current operational limitations. Pressure-assisted crystallization and high-pressure cycling, for instance, improve the crystallinity and stability of HOIPs.^8–12^ Moreover, physical pressure reduces defect density through increased activation barriers for ion migration, enhancing optoelectronic performance.^13^

Three-dimensional HOIPs adopt a common ABX_3_ cuboctahedral structure, in which the A-site cavity is occupied by an organic cation encapsulated by a corner-sharing metal-halide framework, held together predominantly by ionic interactions. This configuration gives rise to an inherent softness, where the interplay between molecular motions of the organic cations and the surrounding inorganic octahedra results in a very-rich phase behavior. Understanding the Pressure–Temperature (P-T) phase diagram remains a challenging task even for the simplest HOIPs.^6,14^ Figure 1 summarizes the available data of the archetypal HOIP MethylAmmonium Lead Iodide (MAPbI_3_). Crystallography reveals the existence of three perovskite phases at ambient pressure, namely a cubic phase (α) above 330 K, a tetragonal phase (β) over the range 160–330 K, and an orthorhombic phase (γ) below 160 K.^15^ Transitions between these as a function of temperature are accompanied by an increased ordering of the MA^+^ organic cation upon cooling.^16,17^ Previous spectroscopic and diffraction studies have confirmed the existence of at least two additional phases at higher pressures: a compressed cubic (δ) and a distorted postperovskite (ϵ) phase, both of which emerge prior to the onset of structural decomposition.^18–20^ The latter phases experience a notable reduction in the volume of the inorganic cage,^21^ concomitant with the emergence of maximal (*a* ^+^ *a* ^+^ *a* ^+^) in-phase tilting of the PbI_6_^–^ octahedra.^20^ It is in these maximally compressed situations that *Im*3̅ symmetry arises for all MAPbX_3_ variants (X = Cl, Br, I).^14,22^

**Figure 1:**
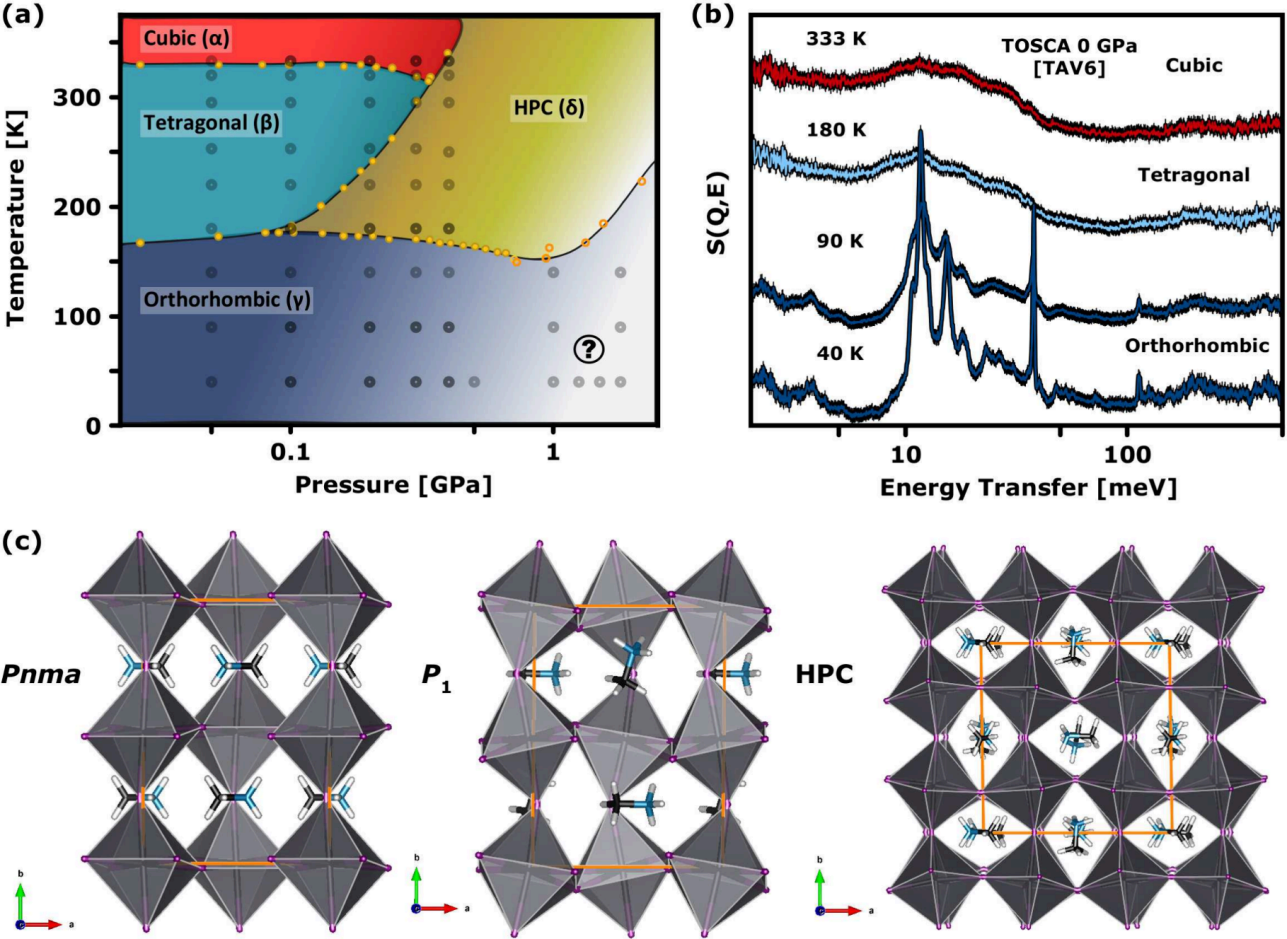
(a) P-T phase diagram of MAPbI_3_. Greek letters (α–δ) denote the phases identified in crystallographic studies. The black lines mark the phase boundaries reported in the literature using dielectric spectroscopy – filled and hollow yellow circles are from refs.,^18,30^ respectively. The gray circles mark the conditions probed in our INS experiments. The question mark (?) highlights the low-temperature, high-pressure region of the phase diagram accessed in the present work. (b) Temperature evolution of the ambient-pressure INS spectra of MAPbI_3_, obtained using the TOSCA TAV6 high-pressure cell (see the Supporting Information (SI) for additional details). The pronounced attenuation of INS signals in both α and β phases is caused by thermal motions, which are suppressed considerably upon entering the γ-phase. (c) Selection of structural models for the γ-phase (*Pnma* and *P*1; *Z* = 4), representing an ordered cation arrangement,^31^ versus the minimal structural model of the δ-phase (HPC; *Z* = 8),^20^ featuring randomly oriented MA^+^ cations inferred from our previous work.^6^

Crystallographic studies on HOIPs primarily focus on the average structures of the inorganic framework. These time- and space-averaged data typically yield limited insight into the spatial arrangement and orientational dynamics of the organic cations.^23^ Spectroscopic probes including Nuclear Magnetic Resonance (NMR) and Inelastic Neutron Scattering (INS) continue to be instrumental at building a more precise microscopic picture of HOIPs.^17,24–26^ Their complementarity to diffraction methods and other techniques stems from their ability to probe the cation dynamics by accessing atomic correlation functions in both space and time, yielding invaluable information about the dynamics of MA^+^ cations and their local environment. In particular, INS offers a high sensitivity to NH···I bonding motifs in HOIPs, since the scattering response is dominated by the large (incoherent) neutron cross section of hydrogen^27–29^ – see Figure 1b and Figure S1.

Information on MAPbI_3_ at low temperatures and pressures above ambient has largely been restricted to the seminal calorimetric study by Onoda-Yamamuro et al.,^32^ and the dielectric spectroscopy investigation of Gesi.^18^ The latter has been extended recently beyond 1 GPa and across a broad temperature range by Chan et al., who identified a new phase boundary above 150 K (see Figure 1a).^30^ Recent synchrotron diffraction studies indicate that the observed dielectric anomaly is of a dynamical nature.^6^ We underscore, however, that this report left the high-pressure, low-temperature end of the phase diagram untouched, thus structural information on MAPbI_3_ at these conditions remains uncharted territory. In addition, isothermal Photo-Luminescence (PL) experiments at high pressures on thin films suggest phase coexistence and a monotonic contraction of the perovskite framework far beyond 1 GPa at low temperatures.^7^ These seemingly contradictory reports leave the precise boundaries of the γ-phase still unknown (note the question mark in Figure 1a). Likewise, the effects of pressurization on the local order around the organic cations remain unexplored given the experimental challenges involved. To fill these gaps in our current understanding of the P-T phase diagram of MAPbI_3_, this work uses high-resolution INS to scrutinize the dynamics of the organic cation in MAPbI_3_ well into the GPa regime. Neutron data are interpreted via the use of first-principles, Harmonic Lattice Dynamics (HLD) calculations performed on a set of structural models of MAPbI_3_ (see Figure 1c and refs 6, 17, 31, and 33 ). These models differ primarily in the orientation of the MA^+^ cations, thus giving rise to distinct hydrogen-bonding (HB) motifs holding the material together. For *Pnma*, there is a strict end-to-end alignment and an evenly distributed NH···I network, whereas symmetry constraints have been relaxed in both *P*1 and HPC models.^6,17,31,33^ The combined use of INS in tandem with first-principles calculations allows us to unravel cation motions and the underlying HB motifs in unprecedented detail as a function of both temperature and pressure, prior to the onset of structural collapse and eventual amorphization of the material.^6,17,31,33^

To this end, we place a focus on exploring the effects of pressurization in the low-energy transfer region of the INS spectra, following the spectroscopic assignments reported in previous works.^17,26,33^ Light-based spectroscopies such as Raman or infrared can access low-energy vibrational features,^34–38^ which are particularly sensitive to local structure. Notwithstanding their merits, disentangling the contributions from the organic cation from the dominant Pb–I vibrations can be a challenging task.^39^ In contrast, INS offers complementary insight owing to its unique sensitivity to hydrogen, making it particularly effective at capturing large-amplitude cation modes, which can be compared directly with computational predictions. As shown in the bottom panel of Figure 2, modes with a prevalent projection on hydrogen include: methyl-group reorientations about the C–N axis coupled to the lead-iodide framework, τ­(CH_3_) at ca. 12 meV; deformations of the HB motif, δ­(N–H···I) at ca. 15–20 meV; and the disrotatory mode τ­(NH_3_), associated with the organic cation and located at ca. 38 meV at ambient pressure. Among these modes, several works to date have shown that τ­(NH_3_) is decoupled from the inorganic lattice.^17,26,38,40^

**Figure 2:**
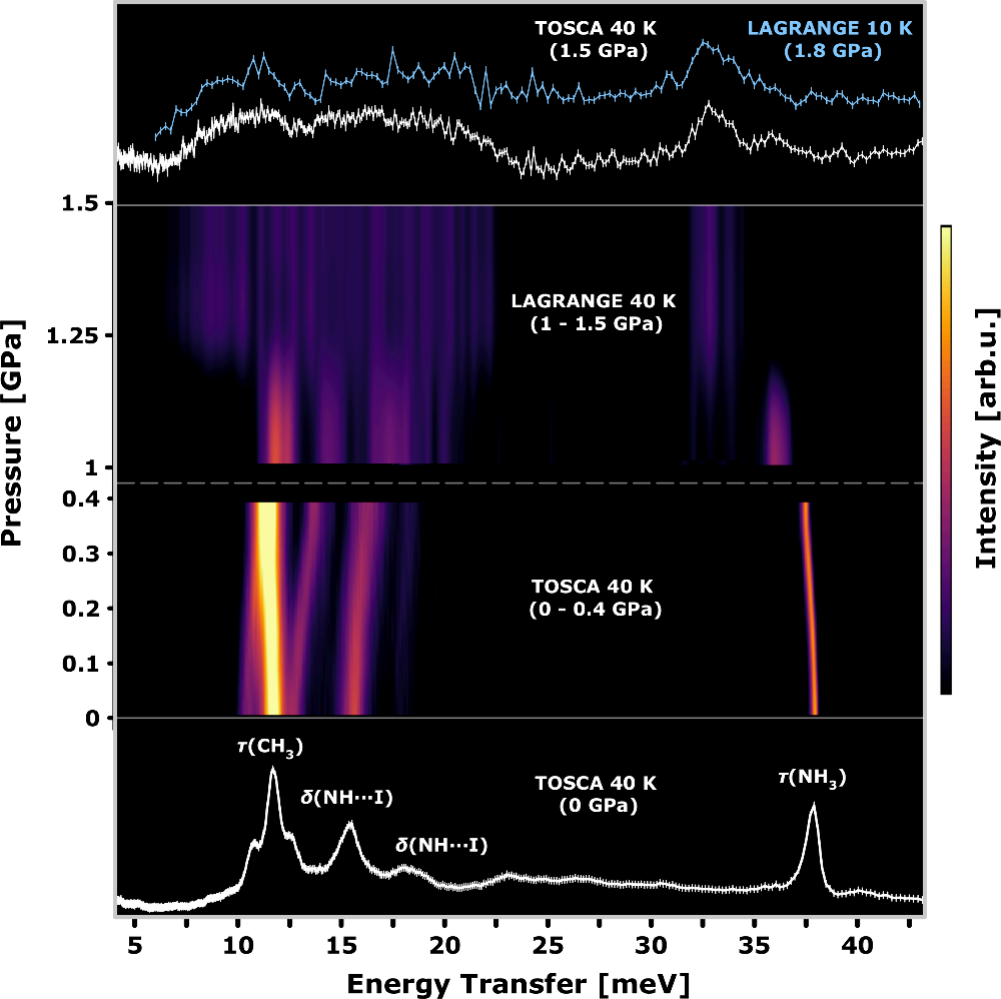
Pressure dependence of the INS spectra at a temperature of 40 K. The two middle panels show heat maps of INS data obtained on the TOSCA and LAGRANGE spectrometers, as explained in more detail in the main text and the Supporting Information. The spectral assignments displayed in the bottom panel have been taken from previous works at ambient pressure.^17,26,33^ The top panel highlights the severe broadening of INS features associated with cation librations above 1.25 GPa.

Figure 2 shows a heat map of INS spectra of MAPbI_3_ up to 1.5 GPa. The measurements were performed at 40 K on TOSCA, utilizing a Ti-6Al-4 V alloy (TAV6) cell – see Figures S1 and S2 for further details. We did not find evidence of helium-gas intercalation when operating in the range 0–0.4 GPa, as the phase boundaries under these conditions remain the same as in our previous study.^6^ A number of spectroscopic works have provided insights on compression at ambient temperature across a broader pressure range,^30,36,37,41–43^ where the position of the N–H stretch mode ν­(N–H) has been taken as a measure of HB strength. Significant red shifts in the position of this mode have been observed for MAPbI_3_ only well beyond 2 GPa,^36,42^ whereas the effects of pressurization on the INS modes associated with HB deformations become quite apparent even at relatively modest pressures. Up to ca. 150 K, the spectra exhibit sharp vibrational features, indicating hindered cation dynamics and a highly directional alignment of the MA^+^ cation.^17^ These results confirm the onset of cation ordering following both the β → γ and δ → γ phase transitions (see the phase diagram in Figure 1a). Beyond the δ → γ transition at 0.2 GPa, the average structure can be described with *Pnma* symmetry, as confirmed by high-pressure neutron diffraction.^6^ Below 1 GPa, the system undergoes compression mainly through octahedral tilting. The δ­(NH···I) deformation modes undergo a continuous blue shift in energy of ca. 3% relative to ambient pressure, whereas the disrotatory τ­(NH_3_) mode shows the opposite trend. The δ­(NH···I) modes act so as to disrupt the HB equilibrium geometry by altering both its distance and angle. At the same time, the softening of the disrotatory τ­(NH_3_) mode reflects a shallower potential energy landscape along a coordinate transverse to the HB, facilitating its destabilization with pressure. In addition, the τ­(CH_3_) mode associated with methyl-group reorientations and corresponding to the most intense spectral features in the INS data, is hardly sensitive to pressure. In line with previous findings,^44^ this behavior indicates that the methyl groups are insensitive to cell contraction upon pressurization and that they play a marginal role in structural stabilization.

In order to access the unexplored region of the P-T phase diagram highlighted in Figure 1a, we capitalized from the new clamp cells available on both LAGRANGE and TOSCA.^45,46^ Figure S2 demonstrates that the backgrounds introduced by these novel sample environments are manageable and can be readily subtracted from the total signal, thereby revealing the dynamics of the MA^+^ cations, as depicted in the two upper panels of Figure 2. At a temperature of 40 K, a pressure of 1.25 GPa leads to an overall (inhomogeneous) broadening of INS features, concomitant with a decrease in spectral intensities. The peak associated with the disrotatory τ­(NH_3_) mode also undergoes a reduction of its intensity and a steep downward shift down to ca. 32 meV. Given the cryogenic conditions probed by our INS experiments, these spectroscopic features do not seem to arise from dynamical correlations like the ones found in ref 47. Instead, we find qualitative similarities between the behavior of MAPbI_3_ above 1 GPa and that previously reported for the orientationally disordered glassy phases of FAPbI_3_ and MAPbI_3_FA.^26,48,49^ In these structures, the organic cations are spatially disordered and do not arrange themselves in preferential directions like in the γ-phase of MAPbI_3_. Nonetheless, while the existence of nonperovskite phases in MAPbI_3_ has been widely discussed in the literature due to their known occurrence in other HOIPs,^50^ there is no solid evidence supporting this hypothesis at temperatures as low as 10 K.^6^

To gain further insights into the γ-phase, we performed first-principles, HLD calculations using the PBEsol-D4 ^51,52^ approximation to the exchange-correlation energy, selected according to previous benchmarks presented in refs 31 and 33 . This phase was modeled using the three structural candidates shown in Figure 1c. These candidates differ substantially in the local environments surrounding the MA^+^ cations: *Pnma* corresponds to maximally constrained NH···I bonds, also exhibiting the shortest HB distance, while both *P*1 and HPC represent two cases where this condition is relaxed, featuring looser bonding configurations which result in three inequivalent HBs connecting the organic and inorganic sublattices. These differences in the local environments have been instrumental to reproduce quantitatively the INS response of MAPbI_3_ based on the underlying VDoS.^17,31^ Figures 3a and 3b display the representative phonon band structures calculated at 0 and 2 GPa for the structural models under scrutiny. The evolution of INS features with pressure is given by the heat maps shown in Figure 3c. The HPC model is distinctly different from the other two as it corresponds to a network of uncorrelated organic cations embedded in a maximally in-phase tilted (*a* ^+^ *a* ^+^ *a* ^+^) inorganic framework, resulting in no long-range orientational order. This structural distinction is clearly manifested by a broader distribution of calculated energies for the disrotatory τ­(NH_3_) feature, highlighting the multiplicity of cation environments in HPC compared to the more ordered *Pnma* and *P*1 phases (cf. Figure 3). At ambient pressure, the *P*1 model outperforms *Pnma*, yet Figures 2 and 3 reveal that it falls quite short at accounting for the observed pressure-induced spectral shifts. The presence of imaginary modes in Figure 3b indicates that the *P*1 framework is also mechanically unstable beyond 1.5 GPa. The phonon modes of both HPC and *Pnma* models, on the other hand, are positive throughout the entire pressure range. We further studied the thermodynamic stability of these structures in terms of the Gibbs free energy. Figure S3 shows that both HPC and *Pnma* models are energetically favored relative to *P*1 at 50 K. These results are consistent with previous benchmarks,^31^ yet at the same time they should be viewed with caution, as the computed free-energy differences fall within the bounds of chemical accuracy (∼1 kcal/mol).

**Figure 3:**
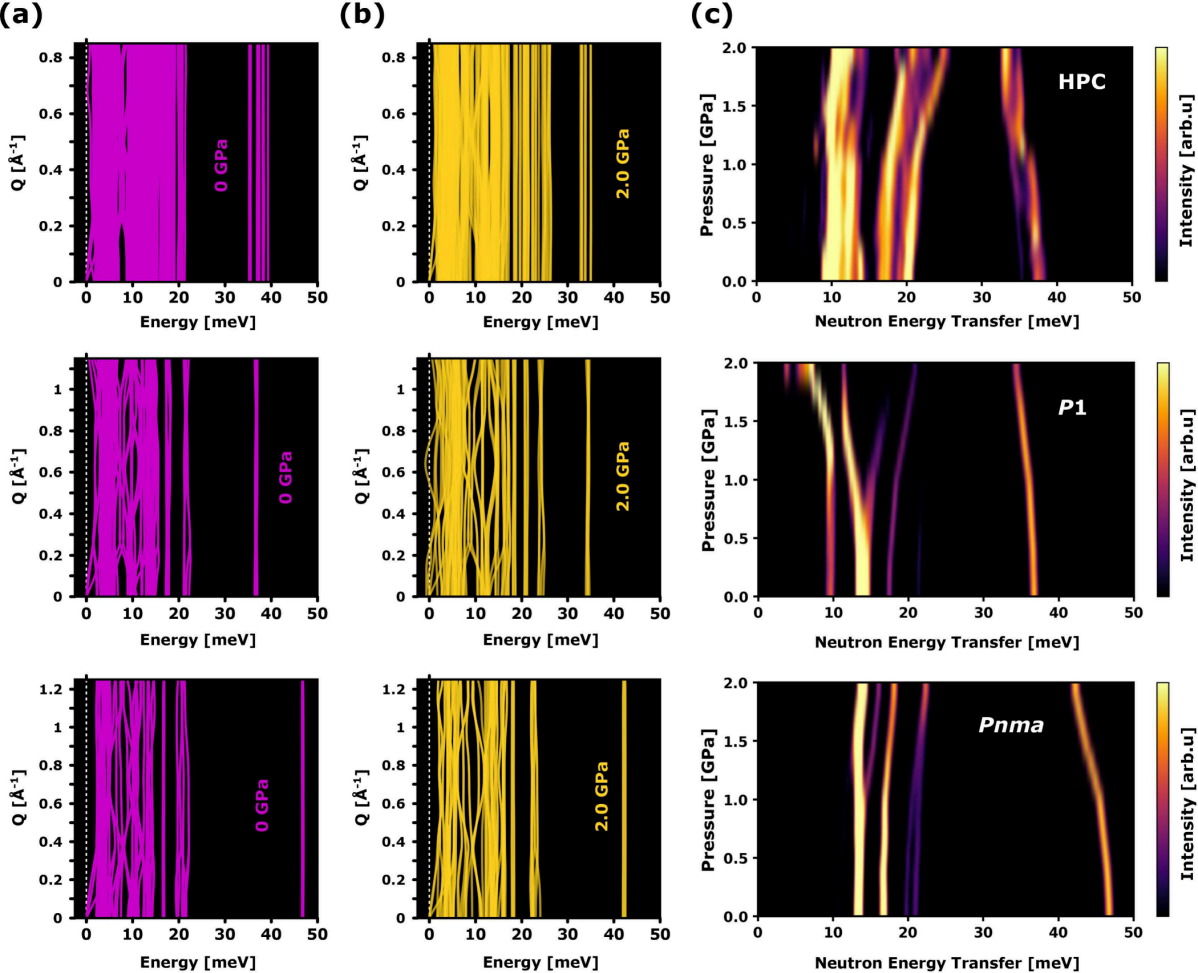
Panels (a) and (b), from top to bottom: calculated phonon-dispersion relations of the HPC, *Pnma* and *P*1 models of MAPbI_3_ under no external pressure (pink) and 2 GPa (dark yellow). (c) Predicted INS response for the aforementioned models as a function of pressure, calculated with AbINS.^53^

Figure 4a offers a more detailed examination of the predictive capabilities of the calculations, with a particular emphasis on the spectral region surrounding the disrotatory τ­(NH_3_) mode. The HPC model is the only one among the three that reproduces the experimentally observed envelope in the INS signal at ca. 35 meV. The three shoulders appearing in the computational predictions of HPC are a consequence of its finite size (*Z* = 8), yet this minimalistic model seems to be sufficient to capture the essential features of the observed INS spectrum. We note that the *P*1 structure can reproduce fairly well the observed spectral shift with pressure, whereas *Pnma* predicts a substantially stiffer τ­(NH_3_) mode and, as a consequence, a harder material. To elucidate the origin of the three distinct INS features of the HPC model, closer inspection of the local environment surrounding the cations is required. Figure 4b highlights the differences between the strong degree of cation disorder characteristic of the HPC model and the network of aligned MA^+^ cations, resulting in the singlet shown in panel [Fig fig4]a for *Pnma* and *P*1 structures.

**Figure 4:**
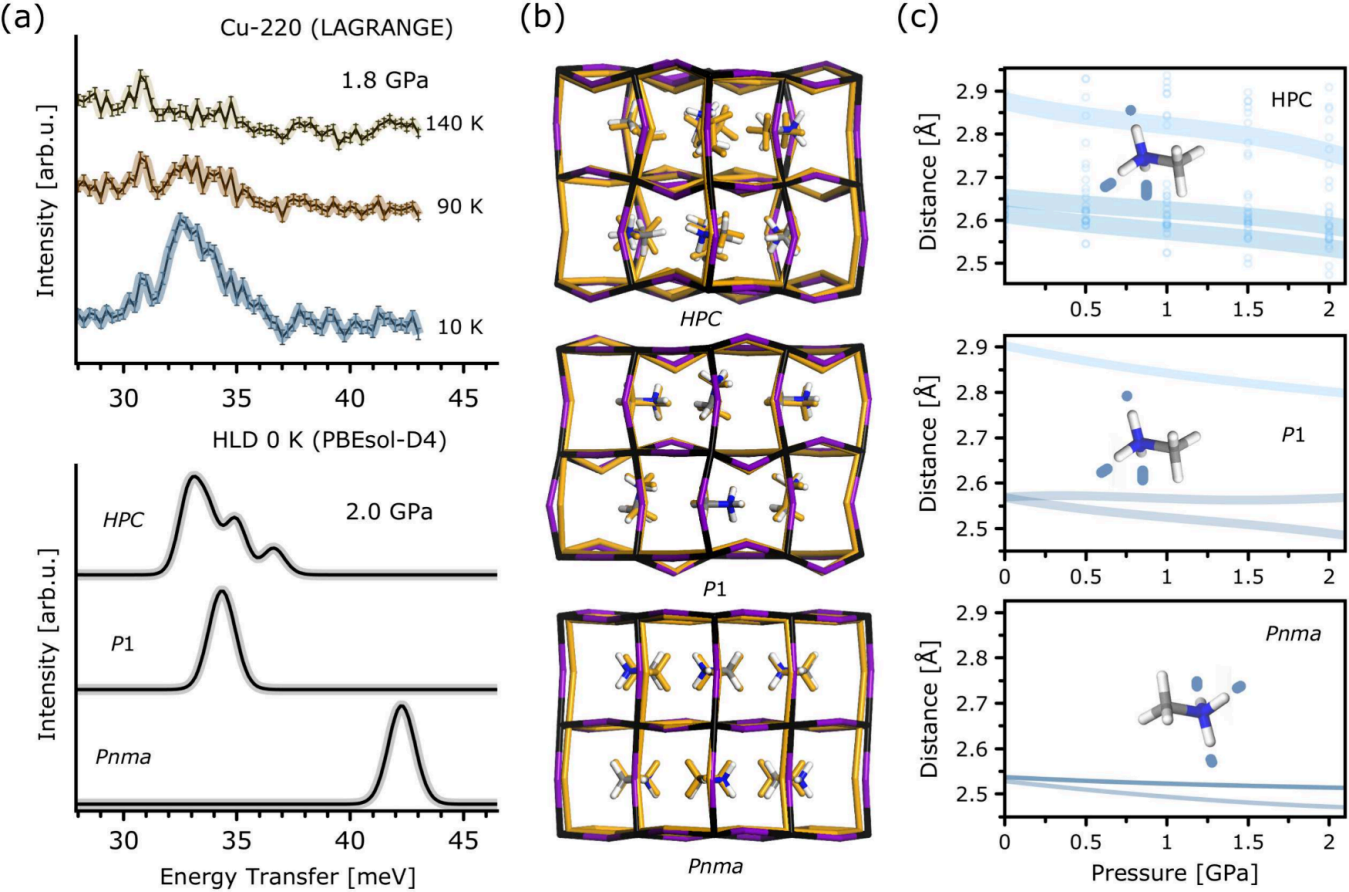
(a) Experimental (top) and computational (bottom) INS spectra at the highest pressures explored in this work. The bottom panel includes predictions for the three structural models discussed in the main text. (b) Superimposed structures from the first-principles calculations at 0 (conventional color scheme) and 2 (yellow) GPa. (c) NH···I bond lengths for each model as a function of pressure. Translucent markers in HPC indicate the distribution of contacts. Light blue lines are guides to the eye.

The uncorrelated nature of this cation disorder arises from the *a* ^+^ *a* ^+^ *a* ^+^ tilting and the Im3̅ symmetry of the inorganic framework.^20,54^ This space group implies the existence of two distinct types of voids in the crystal lattice,^20^ previously denoted as “spherical” and “dumbbell” voids.^55^ Such a configuration implicitly assumes dynamically averaged MA^+^ cations, which are compatible only with the time- or space-averaged symmetry. Similar considerations apply to the proposed orthorhombic *Immm* hettotype discussed elsewhere.^54^ The hindered cation dynamics lead to a metastable phase beyond 1.25 GPa, in the form of an orientational glass embedded within a maximally tilted *a* ^+^ *a* ^+^ *a* ^+^ inorganic framework (see also the SI). This phase exhibits cation disorder, as it is also seen in its higher-temperature counterparts. This situation departs significantly from the cation-ordered structures that have been discussed in the literature for the γ-phase,^31^ where the MA^+^ units show a high degree of dipolar alignment. From these considerations, we can conclude that the HPC model represents the minimal structure which can account for the ’derailment’ of the organic cations at low temperatures and high pressures. As discussed in the SI, pressurization was performed prior to cooling, thus, at this juncture we can only say that the transition temperature is somewhere between 40 K and room temperature. The metastable nature of this phase is in line with the findings of Pieniążek et al., indicating the stabilization of the cation-ordered γ-phase up to approximately 3 GPa at 40 K.^7^ It is unlikely that this particular scenario could be tackled with success by only considering static calculations of crystallographic models at 0 K, yet its main ingredients seem to be captured by the HPC model considered in the present work. This situation implies the presence of a distribution of HB lengths which is absent in the cation-ordered models (see Figure 4c). This distribution arises from the existence of three distinct contacts, in a similar fashion as in *P*1. While in both *Pnma* and *P*1 there is one contact which remains relatively unaffected by pressurization, the characteristic distances between MA^+^ and the inorganic framework in HPC undergo a monotonic decrease with pressure. We observe that the quantitative agreement with the high-pressure INS data is achieved with a HB configuration of two seemingly equal NH···I bonds and a third, longer HB distance. Armed with these (experimentally validated) computational results, we have also scrutinized the effect of pressurization on the optoelectronic properties of the candidate structural models. The main contributors to the electronic density of states of MAPbI_3_ around the band gap correspond primarily to the 6s and 5p orbitals of lead and iodine, respectively, while the organic cation may be regarded as a spectator.^7,56^ The band gap remains almost constant in the HPC structure with pressure, and *Pnma* and *P*1 exhibit a significant reduction that is not consistent with experimental data. These effects can be linked to the tilting of the inorganic sublattice, with changes in lead–iodine-lead angles playing a significant role. Figure S4 shows that the HPC model qualitatively reproduces the experimental trends found by structural refinement methods.^7^

Our results for MAPbI_3_ can also be placed within the context of other high-pressure Raman and infrared studies in halide perovskites extending well into the GPa range, which have signaled the emergence of pressure-induced disorder.^6,30,36,37,41–43^ In particular, Chan et al.^30^ has proposed that the highly compressed low-temperature phase can be stabilized up to room temperature for all three CH_3_NH_3_PbX_3_ analogs (X = I, Br, Cl), revealing a similar spectral broadening. The progressive loss of long-range order upon further compression of this phase has been found to be reversible and it has been ascribed to microscopic mechanisms such as defect-mediated stress relaxation.^43^ The high-pressure phase identified in the present work may be regarded as its low-temperature counterpart, where in both cases cation orientations are frozen and the dynamics quenched, either by the low temperatures or by the extreme pressures. Its metastable nature is further attested by reversible transitions back to ambient-pressure perovskite phases.^9,21,36^

In summary, we have carried out the first exploration of the low-temperature, high-pressure region of the P-T phase diagram of MAPbI_3_ combining INS and first-principles calculations. Capitalizing from recent advances in sample-environment equipment, we have accessed the GPa range with INS, enabling us to probe in unprecedented detail the effects of pressurization on the local environment of MA^+^ cations. Up to 1 GPa, we observe a gradual shift in the position of low-energy spectral features, and ascribe these to changes in HB topology. Between 1 and 1.25 GPa, the INS response shows marked spectral broadenings. First-principles calculations allow us to interpret these changes as signatures of a qualitatively different local environment for the organic cations, consistent with those of the high-pressure cubic phase as reported earlier.^6^ We find a substantial degree of cation disorder, as evidenced by a broad distribution of NH···I bond lengths, in stark contrast with the paradigmatic cation-ordered structures corresponding to the low-temperature phase at ambient pressure. Our efforts also serve to highlight the emerging opportunities in high-pressure INS alongside other complementary techniques like pair-distribution-function analysis, to explore in detail the complex structural dynamics of other HOIPs and related materials under extreme conditions. With these, pending questions such as the microscopic mechanism for the transformation of MAPbI_3_ into the new low-temperature phase identified in this work might be within reach.

## Experimental and Computational Details

Experiments were performed using the MAPbI_3_ specimen described in previous works.^6,17,31,33,48^ The high-pressure INS experiments were carried out on TOSCA (ISIS Neutron and Muon Facility, Rutherford Appleton Laboratory, UK) and LAGRANGE (Institut Laue Langevin, France).^57–60^ Data reduction and cell subtraction were performed using Mantid.^61^ The theoretical calculations as a function of pressure (0–5 GPa) were performed with the CASTEP code (v24.1),^62^ using the PBEsol functional augmented with Grimme’s D4 semiempirical dispersion corrections.^51,52^ The numerical settings were consistent with those used in our previous studies on MAPbI_3_.^17,26,31^ Further experimental and computational details are given in the SI.

## Supplementary Material



## Notes

Experimental and computational details, additional INS results, and computational insights into phase stability and lattice compression (PDF)
