# Supplementary material for: Molecular Derailment via Pressurization in Methylammonium Lead Iodide
Source: J Phys Chem Lett. 2025 Oct 13;16(42):10906–14. doi: 10.1021/acs.jpclett.5c01832 (PMC12557374; doi:10.1021/acs.jpclett.5c01832)
Supplement: Supplementary file 1 [file jz5c01832_si_001.pdf]

Supplementary Information for Publication Molecular Derailment via Pressurization in Methylammonium Lead Iodide

Pelayo Marin-Villa,[†] Pablo Gila-Herranz,[†] Monica Jimenez-Ruiz,[‡] Alexandre Ivanov,[‡] Jeff Armstrong,[¶] Kacper Drużbicki,[§] and Felix Fernandez-Alonso[*,†,‖,⊥]

†*Centro de Física de Materiales (CFM-MPC), CSIC-UPV/EHU, Paseo de Manuel Lardizabal 5, Donostia,20018, Gipuzkoa, Spain*

‡*Institut Laue Langevin, F-38042 Grenoble, France*

¶*ISIS Neutron and Muon Facility, Rutherford Appleton Laboratory, Didcot, OX11 0QX, United Kingdom*

§*Polish Academy of Sciences, Centre of Molecular and Macromolecular Studies, Sienkiewicza 112, 90-363 Lodz, Poland*

‖*Donostia International Physics Center (DIPC), Paseo de Manuel Lardizabal 4, 20018 Donostia - San Sebastian, Spain*

⊥*IKERBASQUE, Basque Foundation for Science, Plaza Euskadi 5, 48009 Bilbao, Spain*

E-mail: felix.fernandez@ehu.eus

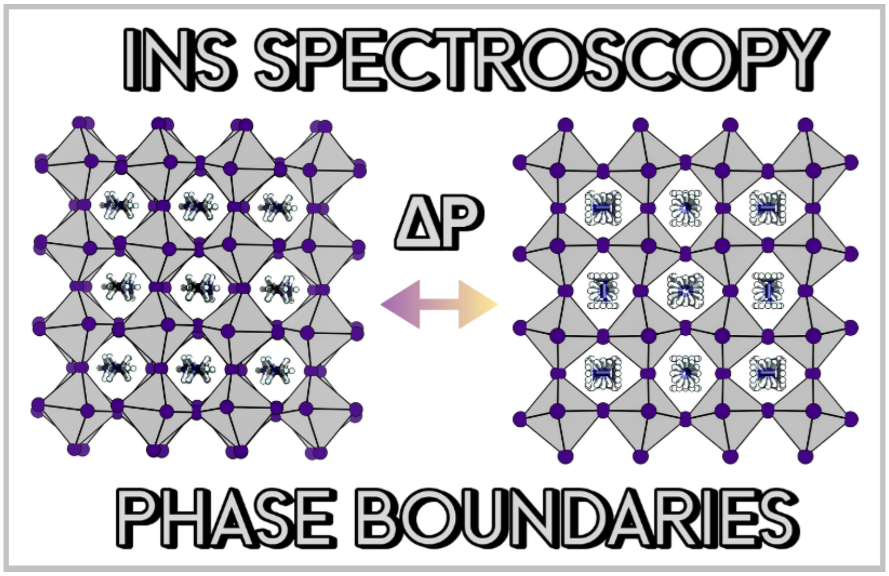

Contents:

S1. Experimental and Computational Details

S1.1. Neutron Spectroscopy under Pressure

S1.2. First-principles Calculations

S2. Additional INS Results

S3. Computational Insights into Phase Stability and Lattice Compression

S1. Experimental and Computational Details

S1.1. Neutron Spectroscopy under Pressure

Inelastic Neutron Scattering (INS) experiments were performed using the same $MAPbI_3$ specimen as in our previous works (CAS No. 69507-98-8, purity >99%; Xi'an Polymer Light Technologies).[1–5] These were carried out on the TOSCA[6–10] and IN1-LAGRANGE[11] spectrometers at the ISIS Pulsed Neutron & Muon Source, Rutherford Appleton Laboratory (UK) and the Institut Laue-Langevin (France), respectively. Measurements up to 0.4 GPa were only performed on TOSCA, on a *ca.* 8 g of powder sample evenly distributed in an aluminum sachet, which was then wrapped around a cylinder and placed inside a Ti-6Al-4V-alloy (TAV6) gas-pressure cell (⌀7 mm). The pressure was controlled externally using a Helium intensifier connected to the cell in order to maintain it constant while changing the temperature. After the cell was sealed, it was screwed to a centerstick and vertically inserted into a Closed Cycle Refrigerator (CCR). INS data were collected as a function of temperature at well-defined isobars, namely, 1 bar, 0.05, 0.1, 0.2, 0.3, and 0.4 GPa.

For pressures above 1 GPa, approximately 1 g of powder sample was loaded together with a 50/50 mixture of Fluorinert 75 and 77 in the TOSCA and IN1-LAGRANGE clamp cells.[12,13] The use of this specific mixture was motivated by: (1) its proven ability to maintain hydrostatic conditions at gigapascal pressures and low temperatures; and (2) its small contribution to the INS response in comparison with the hydrogen-rich sample.[14] The nominal pressure inside the cell was set at room temperature using a calibrated reference, before being shielded by a Cadmium foil. Afterwards, the cell was screwed to a centerstick and vertically inserted into the CCR. Careful alignment with the beam guaranteed a count rate which allowed to resolve spectral features within an hour. While we did not quantify the possible uniaxial stress within the clamp cell, comparison of the spectra with the theoretical predictions indicates that its relative contribution to the INS response minimal. Measurements on IN1-LAGRANGE employed the Si(111), Si(311), and Cu(220) monochromators to

record INS data over the entire spectral range available on the instrument. In all cases, the data were reduced using Mantid.[15]

S1.2. First-principles Calculations

Density Functional Theory (DFT) calculations of the electronic structure and Vibrational Density of States (VDoS) of $MAPbI_3$ were carried out at 0 K over a broad pressure range (0 – 5 GPa) using both static and time-dependent computational methods, employing the structural models of the low-temperature phase introduced in our previous works (*Pnma* and $P1$, both with $Z = 4$).[1,16] The structural model of the High-Pressure Cubic (HPC) phase was constructed based on the room-temperature, high-pressure (2.34 GPa) Single-Crystal X-Ray Diffraction (SCXRD) data reported by Szafrański *et al.*[17] The average structure of the HPC phase of $MAPbI_3$ is described by the cubic, non-polar $Im\overline{3}$ space group, with eight formula units per unit cell ($Z = 8$). Accordingly, the eight MA^+ cations within the HPC structure occupy two distinct Wyckoff sites whose site symmetries exceed the intrinsic symmetry of the molecular cation. This resulting orientational disorder (of at least eightfold multiplicity) cannot be resolved unequivocally.[17] In view of this limitation, cation orientations were obtained from our recent high-pressure *Ab Initio* Molecular Dynamics (AIMD) simulations employing an extended atomistic model with $Z = 64$.[5] The average structure of the eight central MA^+ cations was translated into the maximally tilted ($a^+a^+a^+$) octahedral framework and structurally relaxed further at a given pressure. In the absence of crystallographic data at the P-T conditions investigated in this work, the abovementioned tilting pattern conforms to X-ray data at 120 K and 2 GPa using XRD, conditions similar to ours.[5] The resulting structures were close to orthorhombic, yet with $P1$ global symmetry. At this point, we note the existence of alternative orthorhombic models reported in the literature with $Z = 8$, starting from the *Pnma* structure reported by Chi *et al.*[18] Candidate orthorhombic superstructures with eight molecules per unit cell were investigated recently to elucidate the low-temperature INS response.[1] However, these spectra exhibit narrow features

which are not consistent with experimental data above 1 GPa. In addition, the existence of a metastable orthorhombic *Immm* average structure has been proposed, characterized by a $a^+a^+a^+$ tilt pattern of the PbI_6 octahedra.[19,20] Still, *Immm* symmetry only describes the inorganic sub-lattice, while it implies a disordered or dynamically averaged configuration of the MA^+ cations which is only compatible with the above if defined in a time- or spatially averaged sense.[19]

The theoretical calculations were performed using the solid-state formulation of DFT as implemented in the CASTEP code (v24.1).[21,22] We utilized the revised Perdew-Burke-Ernzerhof functional for solids (PBEsol),[23] with the D4 semi-empirical dispersion corrections of Grimme *et al.*[24] Numerical settings were the same as those used in our previous studies on $MAPbI_3$.[1,16,25] A set of hard Norm-Conserving Pseudo-Potentials (NCPPs) described the core electrons. The electronic wave functions were defined using a Plane-Wave (PW) basis set with a kinetic energy cutoff of 900 eV. The NCPPs were generated on-the-fly using PBEsol. A 2×2×2 Monkhorst-Pack (M-P) grid was used to sample the k-point mesh. All structures were optimized at target pressures to minimize residual atomic forces. The convergence criteria in the total energy, Hellmann–Feynman forces, external stress, maximum displacement, and Self-Consistent-Field (SCF) cycles were 1×10^{-12} eV/atom, 1×10^{-5} eV/Å, 0.0001 GPa, 1×10^{-6} Å, and 1×10^{-12} eV/atom, respectively. Following geometry optimization, phonon frequencies and eigenvectors were calculated throughout the first Brillouin zone. Dynamical matrices were constructed via numerical differentiation of the analytical gradients with respect to atomic displacements with an amplitude of 0.01 Å. The non-diagonal supercell method of Lloyd-Williams and Monserrat was employed to reduce the supercell size required to obtain the force constants.[26]

S2. Additional INS Results

Figures S1a and S1b display the variable-temperature INS spectra of $MAPbI_3$ obtained on TOSCA at ambient pressure and 0.4 GPa. Our recent diffraction study, performed with the

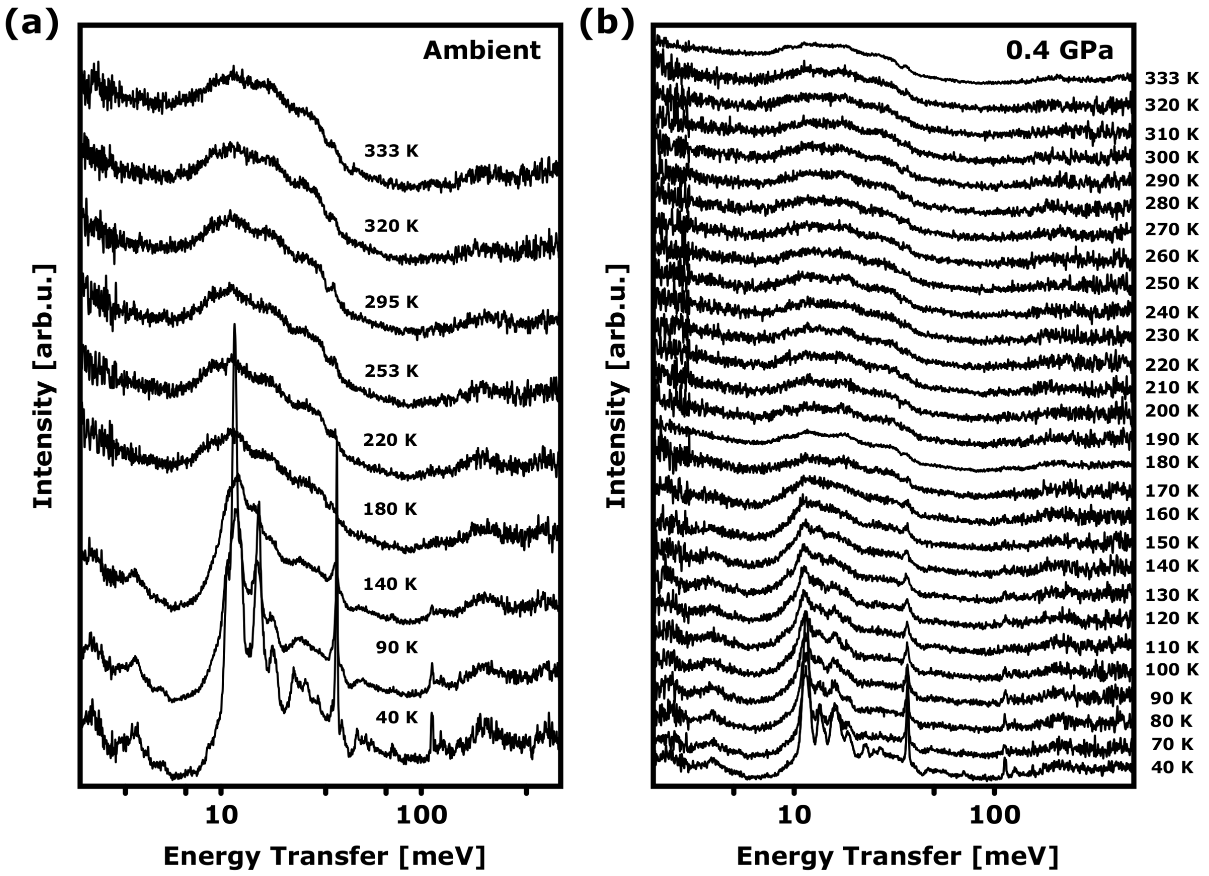

Figure S1: Temperature evolution of INS spectra of $MAPbI_3$ recorded on TOSCA (TAV6 cell) at (a) ambient pressure and (b) along the 0.4 GPa isobar, following heating from base temperature. For clarity, these data have been offset vertically. The high-pressure spectra at 40 K, 180 K, and 333 K, respectively, were acquired with extended counting times of *ca.* 2 hours to improve signal quality.

same sample environment, indicates the presence of three distinct phases at ambient pressure.[5] The cation-ordered γ-phase was found to be thermally stable up to 160 K. A phase transition is then triggered by the onset of the MA^+ reorientation dynamics, leading to a tetragonal β-phase. This structure remains stable up to 330 K, eventually transforming into the cubic α-phase. As shown in Fig. S1a, broad INS spectra relatively insentitive to temperature are obtained at 180 K and above owing to rapid, stochastic reorientations of the MA^+ cations, characteristic of a plastic crystal.[27] In contrast, the cation-ordered γ-phase exhibits sharp and well-defined spectral features as the temperature is lowered, although substantial broadenings can be observed above 60-70 K. Similar trends can be observed along the 0.4

GPa isobar presented in Fig. S1b, following the transition from the low-temperature γ-phase to the HPC (δ)-phase above 150 K.

Figure S2 shows a comparison between the INS spectra measured on TOSCA using different high-pressure sample environments, illustrating that these devices can be used without a significant loss in the INS intensity as compared to standard aluminum cells. Furthermore, comparison of the INS spectra collected with the high-pressure cells at ambient pressure with those reported in previous works indicate that multiple-scattering effects may be regarded as negligible. A closer look at Figs. S1 and S2 confirms that the broad spectral envelopes observed in the high-temperature regime originate from the MA^+ cations rather than from the sample cell.

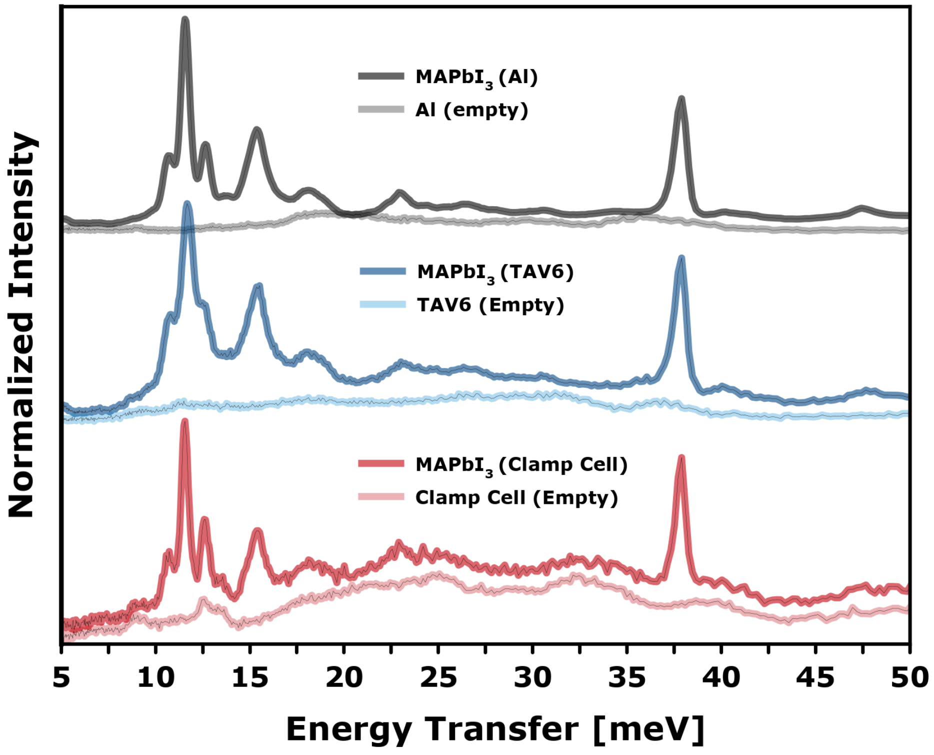

Figure S2: Comparison between the normalized INS signals for $MAPbI_3$ in the different TOSCA cells employed in the present work: standard Al (top); TAV6 (middle); and clamp cell (bottom).

S4. Computational Insights into Phase Stability and Lattice Compression

Within the harmonic approximation, the Gibbs free energy of a system at a given pressure P and temperature T can be written as follows:[1,28]

$$G(P,T) = E_{\mathrm{tot.}} + F_{\mathrm{vib.}}(T) + PV, \tag{1}$$

where $E_{tot.}$ is the total electronic energy at 0 K, $F_{vib.}(T)$ is the vibrational (Helmholtz) free energy, and PV stands for the Pressure-Volume work.[1,28]

Harmonic vibrational energies were obtained for each normal mode j at a given wave vector q using the calculated phonon-dispersion relations (see Section S1). VDoSs were then obtained by integrating over all q-points within the first Brillouin Zone. Using the integrated VDoS, the Helmholtz free energy can be expressed as:[1,28,29]

$$F_{vib.}(T) = E_{ZPE} + F(T) = k_{\mathrm{B}}T \int_0^\infty ln\left[2\sinh\left(\frac{E}{2k_{\mathrm{B}}T}\right)\right] D(E)\, dE \tag{2}$$

where k_B is Boltzmann´s constant and $D(E)$ stands for the VDoS, defined as the number of modes per unit energy. E_{ZPE} is the vibrational zero-point energy and $F(T)$ denotes the temperature-dependent vibrational contribution to the free energy. In this convention, the Helmholtz free energy includes both terms, which can be further expressed as:

$$E_{ZPE} = \frac{1}{2}\int_0^\infty E \cdot D(E)\, dE, \tag{3}$$

and

$$F(T) = k_B T \int_0^\infty ln\left[1 - e^{-E/k_B T}\right] D(E)\, dE \tag{4}$$

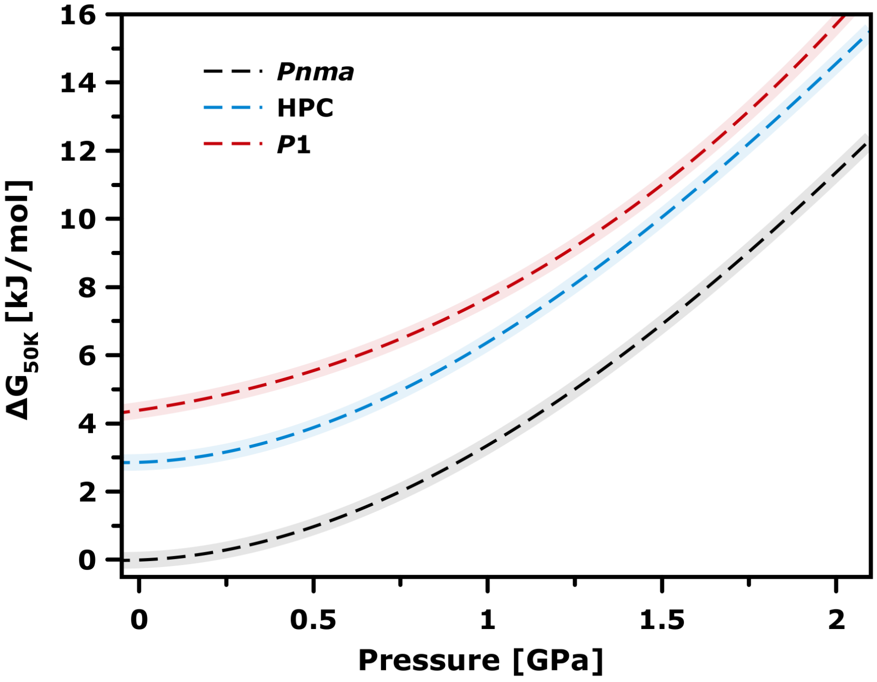

Figure S3: Pressure dependence of the Gibbs free energy at 50 K ($\Delta G_{50\mathrm{K}}$) for the structural models considered in the present work (*Pnma*, HPC, and *P*1).

Figure S3 displays the relative Gibbs free energy per formula unit, calculated at 50 K as a function of pressure. In line with our previous findings,[1,4,5] the *Pnma* phase ($Z = 4$) exhibits the lowest Gibbs free energy at the level of theory presented in this work. The distorted orthorhombic configuration represented by the *P*1 model ($Z = 4$) lies approximately 4 kJ/mol above the ground state, while the HPC model is more stable across the examined pressure range. The observed free energy differences fall within the typical margin of chemical accuracy, beyond the reach of Generalized Gradient Approximation (GGA) methods used in DFT.[30,31] Thus, the relative differences should be interpreted with caution, owing to intrinsic limitations of the PBEsol functional and the dispersion corrections employed in this work. Furthermore, this semi-quantitative assessment is limited by the use of the static harmonic approximation, which does not account for configurational entropy nor mode anharmonicity.[32,33]

Figure S4 presents the pressure dependence of key structural and electronic parameters in $MAPbI_3$, as obtained from the DFT calculations with the PBEsol-D4 functional. Avail–

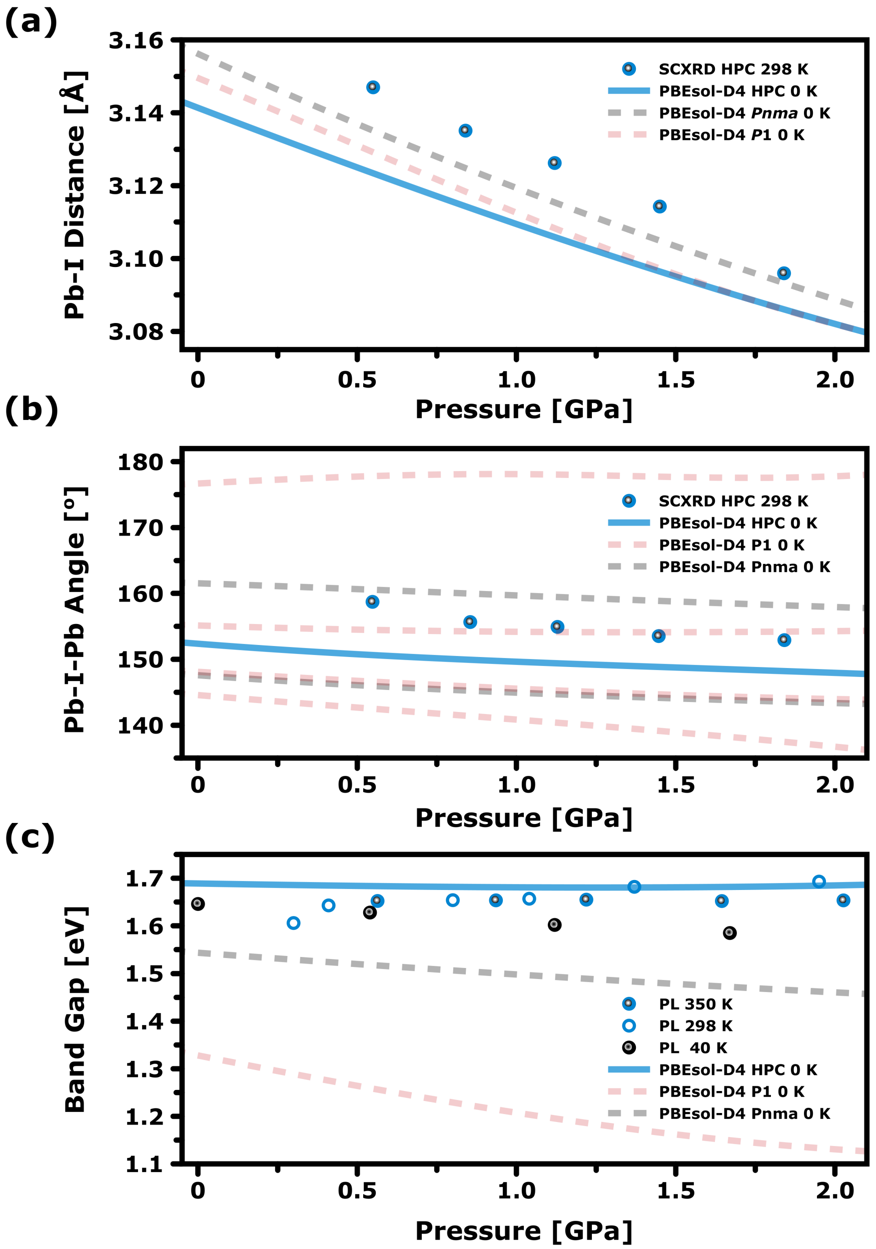

Figure S4: Pressure dependence of the (a) average Pb-I distances; (b) Pb-I-Pb angles; and (c) electronic band gaps, according to HLD calculations (PBEsol-D4) for the structural models considered in the present work (*Pnma*, HPC, and *P*1). Symbols in panels (a) and (b) correspond to room-temperature SCXRD measurements by Szafrański *et al.*[17] Filled symbols in panel (c) are from the PL experiments by Pieniążek and co-workers, [34] recorded for a thin-film specimen at two given isoterms (40 and 350 K). Empty circles are from room-temperature PL measurements on a powder specimen, as reported by Jiang *et al.*[19]

able experimental data are provided for comparison. The Pb-I bond lengths and Pb-I-Pb angles have been taken from the room-temperature SCXRD diffraction data of Szafrański *et*

al.. The evolution of the electronic band gap with pressure corresponds to the laser-induced Photo-Luminescence (PL) studies of Pieniążek *et al.* and Jiang *et al.*.[19,34] A continuous shortening of the Pb–I bond enhances orbital overlap, increasing band dispersion and decreasing the band gap, whereas octahedral tilting entails a reduction of the Pb–I–Pb angle which reduces orbital overlap, consequently widening the associated band gap.[35] In the case of $MAPbI_3$, the competition between these two effects determines the pressure dependence.[36] This structure-property relationship is straightforward for highly symmetric $Im\bar{3}$, where the Pb–I–Pb angles and the bond lengths are nearly uniform. In contrast, it becomes less evident in lower-symmetry models where local distortions, competing octahedral tilts, and cation-driven interactions introduce significant deviations in its inorganic framework. Spin-orbit coupling was not included in the calculations, yet the present model of the HPC phase shows good agreement with the available experimental data.

References

(1) Drużbicki, K.; Gila-Herranz, P.; Marin-Villa, P.; Gaboardi, M.; Armstrong, J.; Fernandez-Alonso, F. Cation Dynamics as Structure Explorer in Hybrid Perovskites The Case of $MAPbI_3$. *Cryst. Growth Des.* **2024**, *24*, 391–404.

(2) Drużbicki, K.; Gaboardi, M.; Fernandez-Alonso, F. Dynamics & Spectroscopy with Neutrons—Recent Developments & Emerging Opportunities. *Polymers* **2021**, *13*, 1440–44.

(3) Marín-Villa, P.; Arauzo, A.; Drużbicki, K.; Fernandez-Alonso, F. Unraveling the Ordered Phase of the Quintessential Hybrid Perovskite $MAPbI_3$ – Thermophysics to the Rescue. *J. Phys. Chem. Lett.* **2022**, *13*, 8422–8428.

(4) Drużbicki, K.; Pinna, R. S.; Rudić, S.; Jura, M.; Gorini, G.; Fernandez-Alonso, F. Unexpected Cation Dynamics in the Low-Temperature Phase of Methylammonium

Lead Iodide: The Need for Improved Models. *J. Phys. Chem. Lett.* **2016**, *7*, 4701–4709.

(5) Marin-Villa, P.; Gaboardi, M.; Joseph, B.; Alabarse, F.; Armstrong, J.; Drużbicki, K.; Fernandez-Alonso, F. Methylammonium Lead Iodide across Physical Space: Phase Boundaries and Structural Collapse. *J. Phys. Chem. Lett.* **2024**, 184–190.

(6) Demmel, F.; McPhail, D.; French, C.; Maxwell, D.; Harrison, S.; Boxall, J.; Rhodes, N.; Mukhopadhyay, S.; Silverwood, I.; Fernandez-Alonso, F.; *et al.* ToF-Backscattering Spectroscopy at the ISIS Facility: Status and Perspectives. *J. Phys. Conf. Ser.* **2018**, *1021*, 012027–5.

(7) Telling, M. T. F.; Campbell, S. I.; Engberg, D.; y Marero, D. M.; Andersen, K. H. Correction: Spectroscopic Characteristics of the OSIRIS Near-backscattering Crystal Analyser Spectrometer on the ISIS Pulsed Neutron Source. *Phys. Chem. Chem. Phys.* **2016**, *18*, 8243–8243.

(8) Demmel, F.; McPhail, D.; Crawford, J.; Maxwell, D.; Pokhilchuk, K.; Garcia-Sakai, V.; Mukhopadyay, S.; Telling, M.; Bermejo, F.; *et al.*, F. F.-A. Opening the Terahertz Window on the OSIRIS Spectrometer. *EPJ Web Conf.* **2015**, *83*, 03003–4.

(9) Telling, M. T. F.; Andersen, K. H. Spectroscopic Characteristics of the OSIRIS Near-backscattering Crystal Analyser Spectrometer on the ISIS Pulsed Neutron Source. *Phys. Chem. Chem. Phys.* **2005**, *7*, 1255–1261.

(10) Demmel, F.; Perrichon, A.; McPhail, D.; Luna Dapica, P.; Webb, N.; Cook, A.; Schooneveld, E.; Boxall, J.; Rhodes, N.; Fernandez-Alonso, F.; *et al.* Silver Jubilee for the OSIRIS Spectrometer: Achievements and Outlook. *EPJ Web Conf.* **2023**, *286*, 03005–6.

(11) Ivanov, A.; Jimenéz-Ruiz, M.; Kulda, J. IN1-LAGRANGE – the New ILL Instrument

to Explore Vibration Dynamics of Complex Materials. *J. Phys. Conf. Ser.* **2014**, *554*, 012001–7.

(12) Ivanov, A.; Sadykov, R.; Jiménez-Ruiz, M. **2018**, Proceedings of the Molecular Spectroscopy Science Meeting MSSM2018, Rutherford Appleton Laboratory Technical Report RAL TR–2018–014 (Chilton, 2018). Weblink: epubs.stfc.ac.uk/work/40650049 (accessed: 2025–09–12).

(13) Armstrong, J.; Wang, X.; Fernandez-Alonso, F. The Unlocking of High-pressure Science with Broadband Neutron Spectroscopy at the ISIS Pulsed Neutron & Muon Source. *Nuc. Inst. and Meth. in Phys. Res. A.* **2022**, *1039*, 167097–6.

(14) Yuan, B.; Mole, R.; Wang, C.-W.; Shumack, A.; White, R.; Li, B.; Tong, X.; Yu, D. Two Pressure Cells for Quasielastic and Inelastic Neutron Scatterings. *EPJ Web Conf.* **2022**, *272*, 02009–4.

(15) Arnold, O.; Bilheux, J. C.; Borreguero, J. M.; Buts, A.; Campbell, S. I.; Chapon, L.; Doucet, M.; Draper, N.; Ferraz Leal, R.; Gigg, M. A.; *et al.* Mantid - Data Analysis and Visualization Package for Neutron Scattering and μSR Experiments. *Nucl. Instrum. Methods Phys. Res. A* **2014**, *764*, 156–166.

(16) Drużbicki, K.; Pinna, R. S.; Rudić, S.; Jura, M.; Gorini, G.; Fernandez-Alonso, F. Unexpected Cation Dynamics in the Low-Temperature Phase of Methylammonium Lead Iodide: The Need for Improved Models. *J. Phys. Chem. Lett.* **2016**, *7*, 4701–4709.

(17) Szafrański, M.; Katrusiak, A. Mechanism of Pressure-Induced Phase Transitions, Amorphization, and Absorption-Edge Shift in Photovoltaic Methylammonium Lead Iodide. *J. Phys. Chem. Lett.* **2016**, *7*, 3458–3466.

(18) Chi, L.; Swainson, I.; Cranswick, L.; Her, J.-H.; Stephens, P.; Knop, O. The Ordered

Phase of Methylammonium Lead Chloride $CH_3ND_3PbCl_3$. *J. Solid State Chem.* **2005**, *178*, 1376–1385.

(19) Jiang, S.; Fang, Y.; Li, R.; Xiao, H.; Crowley, J.; Wang, C.; White, T. J.; Goddard, W. A.; Wang, Z.; Baikie, T.; *et al.* Pressure-dependent Polymorphism and Bandgap Tuning of Methylammonium Lead Iodide Perovskite. *Angew. Chem. Int. Ed.* **2016**, *55*, 6540–6544.

(20) Jiang, S.; Fang, Y.; Li, R.; White, T. J.; Wang, Z.; Baikie, T.; Fang, J. Pressure-Induced Phase Transitions and Bandgap-Tuning Effect of Methylammonium Lead Iodide Perovskite. *MRS Adv.* **2018**, *3*, 1825–1830.

(21) Payne, M. C.; Teter, M. P.; Allan, D. C.; Arias, T.; Joannopoulos, J. D. Iterative Minimization Techniques for Ab Initio Total-energy Calculations - Molecular-Dynamics and Conjugate Gradients. *Rev. Mod. Phys.* **1992**, *64*, 1045–1097.

(22) Clark, S. J.; Segall, M. D.; Pickard, C. J.; Hasnip, P. J.; Probert, M. I. J.; Refson, K.; Payne, M. C. First Principles Methods Using CASTEP. *Z. Kristallogr.* **2005**, *220*, 567–570.

(23) Perdew, J. P.; Ruzsinszky, A.; Csonka, G. I.; Vydrov, O. A.; Scuseria, G. E.; Constantin, L. A.; Zhou, X.; Burke, K. Restoring the Density-Gradient Expansion for Exchange in Solids and Surfaces. *Phys. Rev. Lett.* **2008**, *100*, 136406–4.

(24) Caldeweyher, E.; Mewes, J.-M.; Ehlert, S.; Grimme, S. Extension and Evaluation of the D4 London-Dispersion Model for Periodic Systems. *Phys. Chem. Chem. Phys.* **2020**, *22*, 8499–8512.

(25) Drużbicki, K.; Lavén, R.; Armstrong, J.; Malavasi, L.; Fernandez-Alonso, F.; Karlsson, M. Cation Dynamics and Structural Stabilization in Formamidinium Lead Iodide Perovskites. *J. Phys. Chem. Lett.* **2021**, *12*, 3503–3508.

(26) Lloyd-Williams, J. H.; Monserrat, B. Lattice Dynamics and Electron-phonon Coupling Calculations Using Nondiagonal Supercells. *Phys. Rev. B* **2015**, *92*, 184301–9.

(27) Hehlen, B.; Bourges, P.; Rufflé, B.; Clément, S.; Vialla, R.; Ferreira, A. C.; Ecolivet, C.; Paofai, S.; Cordier, S.; Katan, C.; *et al.* Pseudospin-phonon Pretransitional Dynamics in Lead Halide Hybrid Perovskites. *Phys. Rev. B* **2022**, *105*, 024306–13.

(28) Dove, M. T. *Introduction to Lattice Dynamics*; Cambridge University Press, 1993.

(29) Baroni, S.; de Gironcoli, S.; Dal Corso, A.; Giannozzi, P. Phonons and Related Crystal Properties from Density-functional Perturbation Theory. *Rev. Mod. Phys.* **2001**, *73*, 515–562.

(30) Braeckevelt, T.; Goeminne, R.; Vandenhaute, S.; Borgmans, S.; Verstraelen, T.; Steele, J. A.; Roeffaers, M. B. J.; Hofkens, J.; Rogge, S. M. J.; Van Speybroeck, V. Accurately Determining the Phase Transition Temperature of $CsPbI_3$ via Random-Phase Approximation Calculations and Phase-Transferable Machine Learning Potentials. *Chem. Mater.* **2022**, *34*, 8561–8576.

(31) Bokdam, M.; Lahnsteiner, J.; Ramberger, B.; Schäfer, T.; Kresse, G. Assessing Density Functionals Using Many Body Theory for Hybrid Perovskites. *Phys. Rev. Lett.* **2017**, *119*, 145501–5.

(32) Fransson, E.; Wiktor, J.; Erhart, P. Phase Transitions in Inorganic Halide Perovskites from Machine-Learned Potentials. *J. Phys. Chem. C* **2023**, *127*, 13773–13781.

(33) Dutta, S.; Fransson, E.; Hainer, T.; Gallant, B. M.; Kubicki, D. J.; Erhart, P.; Wiktor, J. Revealing the Low Temperature Phase of $FAPbI_3$ using a Machine-Learned Potential. *J. Am. Chem. Soc.* **2025**, DOI: 10.1021/jacs.5c05265.

(34) Pieniążek, A.; Dybała, F.; Polak, M. P.; Przypis, L.; Herman, A. P.; Kopaczek, J.;

Kudrawiec, R. Bandgap Pressure Coefficient of a $CH_3NH_3PbI_3$ Thin Film Perovskite. *J. Phys. Chem. Lett.* **2023**, *14*, 6470–6476.

(35) Smith, I. C.; Smith, M. D.; Jaffe, A.; Lin, Y.; Karunadasa, H. I. Between the Sheets: Postsynthetic Transformations in Hybrid Perovskites. *Chem. Mater.* **2017**, *29*, 1868–1884.

(36) Celeste, A.; Capitani, F. Hybrid Perovskites under Pressure: Present and Future Directions. *J. Appl. Phys.* **2022**, *132*, 220903–15.
